# Contribution to dose in healthy tissue from secondary target fragments in therapeutic proton, He and C beams measured with CR-39 plastic nuclear track detectors

**DOI:** 10.1038/s41598-019-39598-0

**Published:** 2019-03-06

**Authors:** Satoshi Kodaira, Hisashi Kitamura, Mieko Kurano, Hajime Kawashima, Eric R. Benton

**Affiliations:** 1 Radiation Measurement Research Team, National Institute of Radiological Sciences, National Institutes for Quantum and Radiological Science and Technology, Chiba, Japan; 2 Department of physics, Oklahoma State University, Stillwater, OK United States

## Abstract

The linear energy transfer (LET) spectrum, absorbed dose and dose equivalent from secondary particles of LET_∞_H_2_O ≥15 keV/μm deposited within the plateau of the Bragg curve in primary particle-induced nuclear target fragmentation reactions in tissue during proton and heavy ion radiotherapy were measured using CR-39 plastic nuclear track detectors and analyzed by means of atomic force microscopy. It was found that secondary target fragments contributed 20% to dose equivalent for primary protons (157 MeV), 13% for primary helium ions (145 MeV/n) and 4% for primary carbon ions (383 MeV/n), respectively. Little research has been done on the contribution from these particles to primary given dose. The smaller contribution measured for energetic carbon ion beams compared to proton beams can be considered an advantage of carbon ion radiotherapy over proton radiotherapy.

## Introduction

Although the benefits and advantages of ion beam radiotherapy have been well documented^1^, one aspect of heavy ion beam radiotherapy that has received only limited attention is the absorbed dose and dose equivalent from nuclear target fragmentation interactions^2,3^ between the primary protons and heavy ions of the radiotherapy beam, and the constituent heavy nuclei, mostly ^12^C and ^16^O nuclei, of the healthy tissue itself deposited in healthy tissue traversed by the plateau of the Bragg curve. These nuclear interactions can be explained in terms of a two-step pre-equilibrium model^4^ wherein a pre-equilibrium direct reaction phase proceeds a later, slower equilibrium decay phase. During the pre-equilibrium direct reaction phase^5^, an energetic proton or heavy ion projectile of the primary beam interacts directly with a heavy target nucleus, resulting in the knockout of various nuclear constituents including secondary protons and neutrons, and usually leaving the target nucleus in an excited state. This excited nucleus then acts much like a compound nucleus in the compound nucleus reaction model, wherein additional particles including secondary protons and neutrons are emitted or evaporated from the target nucleus in order for the residual target nucleus to de-excite to a lower energy state. As a result of this nuclear evaporation process the residual heavy target nucleus must recoil in the opposite direction to that of the evaporation particles. This heavy recoil has a range of less than one to tens of micrometers and the directional distribution of recoil nuclei will be more or less isotropic due to the fact that the heavy target nucleus reaches energetic equilibrium, i.e. statistically equal distribution of excitation energy among nucleons making up the nucleus, prior to the evaporation/recoil phase. The short range of these heavy recoil nuclei also means that they are of high LET and thus lead to deposition of relatively large, highly localized dose^2,3,6^. It is important to note that while these recoil secondary particles are of short range, this range is on the scale of biological cells^7^.

Because of their short range, heavy target fragment recoil secondaries typically cannot pass from the target volume into a detector. CR-39 plastic nuclear track detector (PNTD) read out using atomic force microscopy (AFM) uniquely has the capability to detect these short range recoil particles^8,9^, since the detector itself also is the target volume. The occurred nuclear reaction in CR-39 is close to the situation in tissue material because of similar composition consisting of H, C and O elements^10,11^. AFM is capable of imaging high LET, short range recoil tracks after only a minimal bulk thickness of the detector layer (the bulk etch) is removed by chemical etching.

The cross section of such nuclear target fragmentation interactions occurring in healthy tissue traversed by a proton or heavy ion radiotherapy beam prior to penetration of the treatment volume depends on the charge and energy of the beam, as well as the charges of the different nuclei that constitute healthy tissue^2,12^. Previous work has shown that for proton radiotherapy beams, approximately 20% of the dose equivalent to healthy tissue traversed by the plateau of the Bragg curve is the result of nuclear target fragmentation interactions^9^. However at present, we know of no treatment planning software that takes into account such nuclear target fragmentation contributions to dose in healthy tissue.

## Materials and Methods

To measure the contribution from proton and heavy ion induced target fragmentation secondaries to the absorbed dose and dose equivalent deposited in the plateau of the Bragg curve, CR-39 PNTD analyzed by means of AFM was used^9^. CR-39 is a cross-linked thermoset polymer having composition and density similar to human tissue and is sensitive to high LET particles of LET_∞_H_2_O ≥~15 keV/μm^13^. When a charged particle with LET_∞_H_2_O ≥15 keV/μm passes through a layer of CR-39, it breaks the chemical bonds of the polymer along its trajectory, leaving what is called a latent damage trail^14^. Following exposure, CR-39 is chemically etched in a highly alkaline solution at elevated temperature for a prescribed period of time. Chemical etching dissolves the polymer. However, it dissolves the material along the latent damage trail at a faster rate than it does the bulk of the polymer. The result after etching is a conical pit, referred to as a nuclear track, in the surface of the PNTD. The size of the elliptical interface of the nuclear track and the post-etch surface of the CR-39 layer is proportional to the LET of the charged particle that produced the original latent damage trail^14,15^. Scanning the post etch surface by means of AFM permits the dimensions of the elliptical nuclear tracks to be measured^16^. Measurement of all the nuclear tracks within a given detector area leads to determination of the particle fluence. Since elliptical track size is proportional to LET, an LET spectrum and integrated values of absorbed dose and dose equivalent can be determined. The measured size of each track is converted to LET by means of an empirically determined detector response function found by measuring tracks of known LET from heavy ion exposures^13,15^. The detector response function was obtained by means of exposure and subsequent readout of CR-39 to beams of known LET particles (i.e. ion beams of various charge and energy) at HIMAC (Heavy Ion Medical Accelerator in Chiba) of National Institute of Radiological Sciences, National Institutes for Quantum and Radiological Science and Tehcnology, Japan. The track fluence of each given LET yields the LET spectrum. Corrections for the CR-39 detection limit for solid-angle dependence and for the intrinsic critical angle dependence of CR-39 on LET are applied in determining LET spectrum^9^. The absorbed dose (*D*) is found from:

1 $${\rm{D}}({\rm{LET}})=\frac{1.602\times {10}^{-9}}{\rho }{\sum }_{i}{F}_{i}\cdot LE{T}_{i}$$

where *F*_i_ is fluence of secondary target fragments with *LET*_i_ and density of material (ρ). The dose equivalent (*H*)^17^ is determined by applying the quality factor (*Q*) as a function of LET according to ICRP 74 publication^18^:

2 $${\rm{H}}({\rm{LET}})=\frac{1.602\times {10}^{-9}}{\rho }{\sum }_{i}{Q}_{i}\cdot {F}_{i}\cdot LE{T}_{i}$$

The threshold sensitivity makes CR-39 ideal for measuring the contribution to absorbed dose and dose equivalent from high LET charge particles produced in nuclear target fragmentation reactions, since the LET of the primary projectiles of the beam in the case of proton and helium beams is less than the minimum LET threshold for track formation in CR-39. This means that, in the plateau of the Bragg curve, primary protons and helium ions do not produce tracks and all tracks visible in the detector must be the result of high LET secondary fragments.

The use of AFM to analyze the exposed and etched layers of CR-39 has a number of advantages. Principal among these is the fact that only a minimum bulk etch layer needs to be removed by chemical etching in order to enlarge the nuclear tracks sufficiently so that they can be accurately detected and imaged. This fact is especially important since the high LET secondary recoil tracks produced in nuclear target fragmentation interactions are typically of extremely short range. In addition, for a charged particle to produce a measurable nuclear track in CR-39 it must possess a range in excess of the bulk etch, i.e. the thickness of the bulk CR-39 removed by the etching process. Given the short range of the target fragment recoil particles, a bulk etch that is as small as possible is desirable. This small bulk etch also has the advantage that the tracks from low LET particles such as primary carbon ions used in carbon radiotherapy remain relatively small and can be easily distinguished from the high LET target fragment tracks. It should be noted that while recoil target fragmentation tracks have extremely short range (on the order of micrometers), such ranges are comparable to the dimensions of biological cells.

## Experimental

CR-39 PNTD (trade name: BARYOTRAK) with dimensions of 19 mm × 19 mm × 0.9 mm, manufactured by Fukuvi Chemical Industry, Japan, was used in all exposures carried out for this work. The detector stacks consisting of two CR-39 layers were irradiated at six different dip angles (*δ* = 90°, 75°, 60°, 45°, 30° and 15°) in order to correct for the solid-angle dependence and critical angle sensitivity of CR-39^9^. The detector stacks were irradiated to 157 MeV proton and 383 MeV/n carbon ion beam at HIMAC. In addition to protons and carbon ions, irradiations were made to beam of 145 MeV/n helium ion beams in order to investigate the nuclear charge dependence of the primary ion beams in target fragmentation reactions. The beam spot size was uniformly spread out to be 10 cm in diameter by the wobbler-scatterer method^19^. The irradiated beam fluence was 1.7 × 10^10^ cm^−2^ for all ion beams.

The beam intensity and total fluence were monitored and controlled with a parallel plate ionization chamber installed in front of CR-39. The irradiated absorbed dose was measured with a Markus ionization chamber^20^. Note that the primary ion beams lose kinetic energy due to the passage of the beam through air, beam monitors and other instruments. There are very few contribution due to the secondary particles generated in the beam path^9,21^. Details of the primary ion beam irradiation is summarized in Table 1.

**Table 1:** Primary ion beam irradiation condition.

Primary ion	Nuclear charge (*Z*)	Incident energy [MeV/n]	*Z/β*	Absorbed dose (*D*_*p*_) [Gy]	LET in water [keV/µm]	Fluence (*F*_*p*_) [cm^−2^]
Proton	1	157	2.0	14.86	0.53	1.7 × 10^10^
Helium	2	145	4.0	62.80	2.25	1.7 × 10^10^
Carbon	6	383	8.5	302.7	10.9	1.7 × 10^10^

The emitted target fragments have very short range, on the scale of several µm. Therefore, the thickness removed by chemical etching was restricted to ~1 µm in order to avoid the over-etching of short range tracks in the CR-39^8,9^. The irradiated CR-39 layers were etched in 7 mol/l sodium hydroxide solution at 70 °C for 30 min, corresponding to the removal of a bulk thickness of 1 µm.

The etched CR-39 surfaces were scanned with AFM (Dimension V; Veeco) equipped with a 125 μm cantilever having a typical tip length of 10 μm and operating in tapping mode (resonant frequency: ~300 kHz). A total area of 62,500 μm^2^ was scanned, consisting of square images of 25 μm and 1024 pixels on a side. The size of the elliptical openings of the tracks were analyzed with a software (HSP-Fit ver.4.47, SEIKO Precision Inc., Japan) employing a precise ellipse fitting algorithm^22^. The bulk etch was measured with AFM as the level difference between the etched surface and the unetched surface^23^.

The measurement resolution of etch pit with AFM is approximately 3%, which causes the uncertainty for LET determination. The another sources of uncertainty comes from the calibration function^13^. According to the uncertainty study in LET specrum^24^, the uncertainty from calibration curve ranges from 1.1% to 9.4% in this work for LET ≥15 keV/µm. The considerable uncertainty comes from statistics in the bin consisting of LET spectrum because, (1) the LET of a track is found as a function of a power law equation^15^, (2) the binning of the LET spectrum^10,25^ is very low resolution (the ratio of LET bin width to the center value of LET~10%), (3) given these two facts, small changes in the measurement of a track, due to AFM or optical microscope analysis or whatever, translate into only small changes in LET, and (4) because of the binning, small changes in LET usually do not translate into any change at all. Therefore, the current measurement method of LET spectrum is not particularly sensitive to the precision of individual track measurements. Instead, overall (random) statistics tend to dominate.

## Results and Discussion

**Figure 1:**
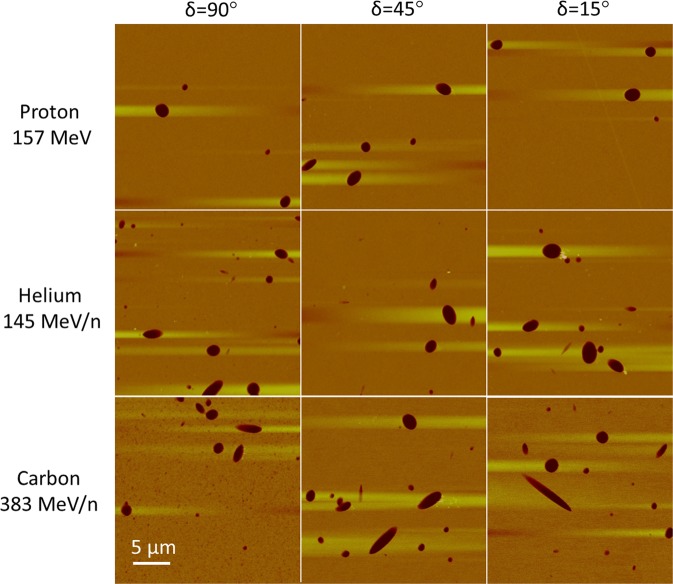
Typical AFM images (25 μm × 25 μm) of etched CR-39 PNTD exposed at three incident angles (*δ* = 90°, 45° and 15°) showing secondary target fragment nuclear tracks from primary proton, helium and carbon ion beams.

Figure 1 shows an array of AFM images measured in CR-39 exposed to proton, helium and carbon ion beams in the plateau of the Bragg curve at three different incident angles. The nuclear tracks are the dark elliptical objects in the images. The lighter stripes lateral to the nuclear tracks are a artifacts of the AFM scanning process. A uniform pattern of very small tracks can be seen in the 383 MeV/n Carbon exposure at δ = 90° (lower left image) that is not seen in any of the other images. These are the tracks from the primary C ions in the beam. They are not visible in the images from the proton and He beams, because the LET of primary ions is too low to register in CR-39. They are not visible in the images of δ = 45° and 15° for the C ions, due to the angular sensitivity of CR-39. All large tracks in these images are due to secondary high LET particles generated inside of ths CR-39 material, not primary particles, since the employed CR-39 does not register tracks from particles of LET less than 15 keV/μm (see Table 1). If CR-39 was sensitive to low LET primary particles, the images would be saturated by a huge number of overlapping tracks due to the high fluence of primary particles (~10^5^ particles/image). Target fragmentation reactions generate secondary particles with various LET (track size is proportional to LET) and emission angle (track ellipticity and focus of ellipse representing the incident angle and direction of the registered particle).

**Figure 2:**
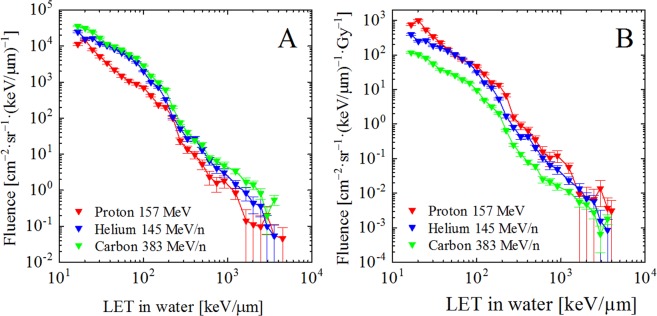
(**A**) LET spectra of secondary target fragments for each primary ion beam and (**B**) LET spectra in which the fluence is normalized to primary beam dose.

**Figure 3:**
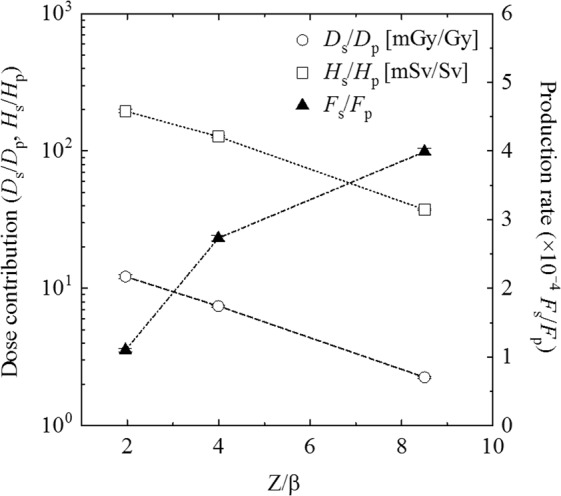
Variations of dose contribution of secondary target fragments to primary irradiation dose (*D*_s_/*D*_p_ and *H*_s_/*H*_p_) and secondary production rate (*F*_s_/*F*_p_) as a function of primary Z/β (Z: nuclear charge and β: velocity).

The LET spectra of secondary target fragments are shown in Fig. 2A. Each LET spectrum follows a continuous power-law curve having several shoulders due to individual target fragment components. Figure 2B shows the LET spectra of secondary target fragments normalized by the absorbed dose of irradiated primary beam (*D*_p_). From the LET spectrum, the absorbed dose (*D*_s_) and dose equivalent (*H*_s_) of secondary particles were determined, as summarized in Table 2, for each primary ion beam. The dose ratios of secondary (*D*_s_ and *H*_s_) to primary (*D*_p_) denote to the dose contribution of secondary particles for primary beam in absorbed dose (*D*_s_/*D*_p_) and dose equivalent (*H*_s_/*H*_p_). The ratio of fluence of secondary target fragments (*F*_s_) to the fluence of primary beam (*F*_p_) denote to the production rate of secondary particles (*F*_s_/*F*_p_). In this work, the incident kinetic energy per nucleon of each primary ion beam was different, so that we employ the ratio of nuclear charge (*Z*) to the velocity (*β*) of the primary ion to facilitate comparison. Figure 3 shows the variation of dose contribution of secondaries (left axis) as a function of primary *Z/β*, as well as the variation of production rates of secondaries (right axis).

**Table 2:** Dose assessment results of secondary target fragments for each primary ion beam.

Primary ion	Absorbed dose (*D*_*s*_) [Gy]	Dose equivalent (*H*_*s*_) [Sv]	Mean quality factor (*Qs* = *H*_*s*_*/D*_*s*_)	Fluence (*F*_*s*_) [cm^−2^]	*D*_*s*_*/D*_*p*_ [mGy/Gy]	*H*_*s*_*/D*_*p*_ [mSv/Gy]	*F*_*s*_ */F*_*p*_
Proton	0.18 ± 0.01	2.91 ± 0.08	16.04 ± 0.66	(1.93 ± 0.04) × 10^6^	12.19 ± 0.38	195.56 ± 5.23	(1.10 ± 0.03) × 10^−4^
Helium	0.47 ± 0.01	8.01 ± 0.12	17.17 ± 0.39	(4.75 ± 0.07) × 10^6^	7.43 ± 0.12	127.46 ± 1.96	(2.73 ± 0.04) × 10^−4^
Carbon	0.68 ± 0.01	11.32 ± 0.15	16.66 ± 0.35	(6.62 ± 0.08) × 10^6^	2.25 ± 0.04	37.41 ± 0.50	(3.99 ± 0.05) × 10^−4^

In accounting for the contribution from nuclear target fragmentation interactions to absorbed dose (*D*_s_/*D*_p_) and dose equivalent (*H*_s_/*H*_p_) in the plateau of the Bragg curve of the proton, helium, and carbon beams, competing phenomena must be considered. First, as seen in Fig. 3, the production rate (*F*_s_/*F*_p_) increases with increasing *Z/β* of the primary projectile particles in the beam. Consequently, the fluence of carbon ion-induced target fragments is four times greater than that of proton-induced target fragments. This observation can be explained by a combination of the *Z* and energy dependences of the nuclear cross-sections for such interactions. Second, in order to deliver a particular therapeutic dose (e.g. the Gray fraction), the fluence of primary particles decreases with increasing *Z* of the primary projectile particle (see Fig. 2B and Table 2). This is largely due to the *Z*^2^ dependence of the Bethe-Bloch stopping power formula^26^. That is, for a given dose fraction, a smaller primary carbon fluence is needed than primary proton fluence. This, of course, is one of the oft–quoted advantages of carbon beam therapy over proton beam therapy.

The net result of these two competing processes can be seen in the measurements of normalized absorbed dose and dose equivalent for proton, helium, and carbon beams listed in Table 2 and shown in Fig. 3. Although the nuclear target fragmentation cross-section for carbon is higher than that for protons, this is more than offset by the *Z*^2^ dependence of the LET such that the contribution to absorbed dose in the plateau of the Bragg curve is 5.4 times lower for carbon ions than for protons and 1.6 times lower for helium ions than for protons. This observation carries over to the dose equivalent contribution of target fragments: 5.2 times lower for carbon ions versus protons and 1.5 times lower for helium ions versus protons. This result can be considered an additional advantage of carbon and helium ion therapy over more established proton beam radiotherapy.

It should be noted that secondary neutrons are produced in nuclear target fragmentation reactions and their contributions to absorbed dose and dose equivalent outside the treatment volume were not assessed in this study.

## Conclusion

The LET spectrum, absorbed dose and dose equivalent resulting from nuclear target fragmentation secondaries of LET_∞_H_2_O ≥15 keV/μm in primary 157 MeV proton, 145 MeV/n helium and 383 MeV/n carbon beam interactions with tissue equivalent material were measured using CR-39 analyzed by AFM. In the plateau of the Bragg curve, the production rate (the ratio of secondary target fragment fluence per primary proton fluence in the beam) and the dose contribution (the ratio of secondary target fragment dose per primary proton dose)were found to be 1.1 × 10^−4^ and 1.2% to absorbed dose and 20% to dose equivalent, respectively. For the primary helium beam, the production rate and dose contribution of secondary particles were found to be 2.7 × 10^−4^ and 0.7% to absorbed dose and 13% to dose equivalent, respectively. For the primary carbon beam, the production rate and dose contribution of secondary particles were found to be 4.0 × 10^−4^ and 0.2% to absorbed dose and 4% to dose equivalent, respectively. These results illustrate the fact that the lower fluence of primary carbon ions versus primary protons needed to deliver a given dose more than compensates for the larger cross section for nuclear fragmentation interactions possessed by carbon ions versus protons traversing a tissue equivalent medium. The results from the helium beam measurements lie between those for the proton and carbon ion beams.

Even though no commonly used treatment planning software currently takes nuclear target fragmentation interactions into account, these results, especially for protons, illustrate that nuclear interactions in tissue in the plateau of the Bragg curve make a significant contribution to dose equivalent. The additional dose equivalent due to the secondary target fragments produced by therapeutic beams, even the low contribution from carbon ion beams, should be assessed from the viewpoint of the radiation protection of the patient. The lower contribution nuclear secondary particles from carbon ions as compared to protons can be considered an advantage of carbon ion radiotherapy over proton radiotherapy.

This type of measurement is not only relevant to radiation protection in cancer therapy, but also in space radiation dosimetry and protection, since 99% of the space radiation environment consists of energetic protons and helium nuclei^27^. Some verification of secondary particles with CR-39 were reported by measuring stopping particles in the detector^28–30^. The type of ground-based experiment reported here are useful for investigating the dose contribution of secondary target fragmentation produced by space radiation to the absorbed dose and dose equivalent received by space crews.

## Data Availability

All relevant data are within the paper.
